# Seasonal Changes in Hematological Parameters in House Sparrows of Subtropical Pakistan

**DOI:** 10.1093/iob/obad027

**Published:** 2023-07-26

**Authors:** S Nimra, A R Kayani, M Irfan, M S Ahmed

**Affiliations:** Department of Biology, Pir Mehr Ali Shah Arid Agriculture University, Rawalpindi, Pakistan; Department of Zoology, Wildlife and Fisheries, Pir Mehr Ali Shah Arid Agriculture University, Rawalpindi, Pakistan; Department of Zoology, Wildlife and Fisheries, Pir Mehr Ali Shah Arid Agriculture University, Rawalpindi, Pakistan; University Institute of Biochemistry and Biotechnology, Pir Mehr Ali Shah Arid Agriculture University, Rawalpindi, Pakistan

## Abstract

House sparrow is a globally adaptive bird. The way this creature adapted to all areas of the world, having different selection pressures, is interesting to understand. The present study is focused on seasonal changes, having different selection pressures and how it is adapted to these changes and whether hematological flexibility plays a role in this success. House sparrow's adaptations in the same area, during different seasons, have been studied in a sub-tropical area, Potohar, Pakistan. We used hematological parameter analysis for this purpose. Blood samples were collected from Sparrows in winter, spring, and summer and analyzed for some hematological parameters. White blood cells (WBCs) were higher in spring and summer which may relate to mating promiscuity. Sparrows were more stressed in summer. The Red blood cells (RBCs) and hematocrit (Hct) were greater in summer. Mean corpuscular volume (MCV) is lower in summer. This may have an adaptation to cope with high stress in summer as small-size RBCs increase gaseous exchange. Platelets were not affected by season or gender. Mean corpuscular volume and Mean corpuscular hemoglobin (MCH) are positively correlated with each other. Red blood cells, hemoglobin (Hb) and MCV were higher in males during the spring season perhaps as an adaptation to energetic activities during spring like mating calls and search for nesting sites. White blood cells remained the same in both genders in summer and winter, and effected in spring may be related to the mating system. Behavioral state is linked with physiological states that shows tradeoff and life history traits. This study is a small effort to know this incredible species. We can work further in different parts of the world to explore different aspects of it.

## Resumen

El gorrión común es un ave que se adapta globalmente. La forma en que esta criatura se adaptó a todas las áreas del mundo, teniendo diferentes presiones de selección, es interesante de entender. El presente estudio se centra en los cambios estacionales, teniendo diferentes presiones de selección y cómo se adapta a estos cambios y si la flexibilidad hematológica juega un papel en este éxito. Las adaptaciones de House Sparrow en la misma área, durante diferentes estaciones, se han estudiado en un área subtropical, Potohar, Pakistán. Utilizamos el análisis de parámetros hematológicos para este propósito. Se recogieron muestras de sangre de gorriones en invierno, primavera y verano y se analizaron algunos parámetros hematológicos. Los glóbulos blancos (WBC) fueron más altos en primavera y verano, lo que puede relacionarse con la promiscuidad de apareamiento. Los gorriones estaban más estresados ​​en verano. Los glóbulos rojos (GR) y el hematocrito (Het) fueron mayores en verano. El volumen corpuscular medio (MCV) es menor en verano. Esto puede tener una adaptación para hacer frente al alto estrés en verano, ya que los glóbulos rojos de pequeño tamaño aumentan el intercambio gaseoso. Las plaquetas no se vieron afectadas por la estación o el género. El MCV y la hemoglobina corpuscular media (MCH) se correlacionan positivamente entre sí. Los glóbulos rojos, la hemoglobina (Hb) y el MCV fueron más altos en los machos durante la temporada de primavera, tal vez como una adaptación a las actividades energéticas durante la primavera, como llamadas de apareamiento y búsqueda de sitios de anidación. Los glóbulos blancos se mantuvieron iguales en ambos sexos en verano e invierno, y los efectuados en primavera pueden estar relacionados con el sistema de apareamiento. El estado de comportamiento está relacionado con estados fisiológicos que muestran rasgos de compensación y de historia de vida. Este estudio es un pequeño esfuerzo por conocer esta increíble especie. Podemos trabajar más en diferentes partes del mundo para explorar diferentes aspectos del mismo.

## Zusammenfassung

Haussperling ist ein global anpassungsfähiger Vogel. Es ist interessant zu verstehen, wie sich diese Kreatur an alle Regionen der Welt angepasst hat und dabei unterschiedlichen Selektionsdruck ausübt. Die vorliegende Studie konzentriert sich auf saisonale Veränderungen, unterschiedliche Selektionsdrücke und wie sie sich an diese Veränderungen anpassen und ob hämatologische Flexibilität für diesen Erfolg eine Rolle spielt. Die Anpassungen des Haussperlings im selben Gebiet zu verschiedenen Jahreszeiten wurden in einem subtropischen Gebiet, Potohar, Pakistan, untersucht. Zu diesem Zweck verwendeten wir eine hämatologische Parameteranalyse.

Im Winter, Frühling und Sommer wurden Blutproben von Sparrows entnommen und auf einige hämatologische Parameter analysiert. Die Zahl der weißen Blutkörperchen (WBCs) war im Frühling und Sommer höher, was möglicherweise auf Promiskuität bei der Paarung zurückzuführen ist. Spatzen waren im Sommer stärker gestresst. Die roten Blutkörperchen (RBCs) und der Hämatokrit (Het) waren im Sommer höher. Das mittlere Korpuskularvolumen (MCV) ist im Sommer niedriger. Dies könnte eine Anpassung an den hohen Stress im Sommer sein, da kleine Erythrozyten den Gasaustausch erhöhen. Die Blutplättchen wurden weder durch Jahreszeit noch durch Geschlecht beeinflusst. MCV und mittleres korpuskuläres Hämoglobin (MCH) korrelieren positiv miteinander. Erythrozyten, Hämoglobin (Hb) und MCV waren bei Männchen während der Frühjahrssaison höher, möglicherweise als Anpassung an energetische Aktivitäten im Frühjahr wie Paarungsrufe und die Suche nach Nistplätzen. Die Leukozytenzahl blieb bei beiden Geschlechtern im Sommer und Winter gleich, und das Auftreten im Frühjahr hängt möglicherweise mit dem Paarungssystem zusammen. Der Verhaltenszustand ist mit physiologischen Zuständen verbunden, die Kompromisse und Merkmale der Lebensgeschichte aufweisen. Diese Studie ist ein kleiner Versuch, diese unglaubliche Art kennenzulernen. Wir können in verschiedenen Teilen der Welt weiterarbeiten, um verschiedene Aspekte davon zu erforschen.

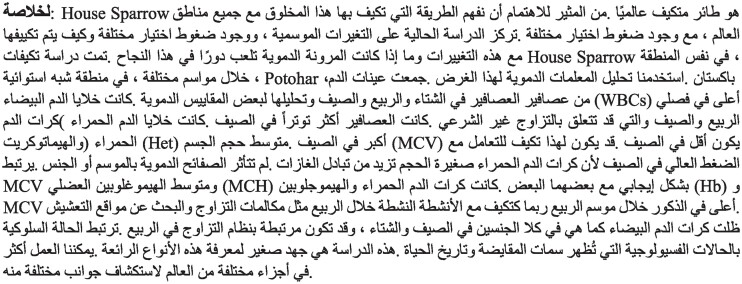

## 摘要

**麻雀是一种具有**全球适应性的鸟类。这种生物适应世界各个地区的方式, 具有不同的选择压力, 理解起来很有趣。本研究的重点是季节变化、不同的选择压力、如何适应这些变化以及血液学灵活性是否在这一成功中发挥作用。巴基斯坦波托哈尔亚热带地区研究了麻雀在同一地区不同季节的适应情况。为此, 我们使用了血液学参数分析。在冬季、春季和夏季从麻雀身上采集血液样本并分析一些血液学参数。白细胞 (WBC) **在春季和夏季**较高, 这可能与交配混乱有关。夏天麻雀的压力更大。夏季红细胞 (RBC) **和血**细胞比容 (Het) 较高。夏季平均红细胞体积 (MCV) 较低。这可能是为了应对夏季的高压力, 因为小尺寸的红细胞增加了气体交换。血小板不受季节或性别的影响。 MCV **与平均**红细胞血红蛋白 (MCH) **呈正相关**。**春季期间**, 雄性的红细胞、血红蛋白 (Hb) **和** MCV 较高, 这可能是为了适应春季期间的活跃活动, 如求偶叫声和寻找筑巢地点。夏季和冬季两性的白细胞保持不变, 春季的影响可能与交配系统有关。行为状态与显示权衡和生活史特征的**生理状**态相关。这项研究只是为了了解这个令人难以置信的物种而做出的小小的努力。我们可以在世界不同地区进一步开展工作, 探索其不同方面。

## Résumé

Le Moineau domestique est un oiseau qui s“adapte à l”échelle mondiale. La façon dont cette créature s“est adaptée à toutes les régions du monde, ayant des pressions de sélection différentes, est intéressante à comprendre. La présente étude se concentre sur les changements saisonniers, ayant différentes pressions de sélection et comment il est adapté à ces changements et si la flexibilité hématologique joue un rôle dans ce succès. Les adaptations du Moineau domestique dans la même zone, à différentes saisons, ont été étudiées dans une zone subtropicale, Potohar, Pakistan. Nous avons utilisé l”analyse des paramètres hématologiques à cette fin. Des échantillons de sang ont été prélevés sur des moineaux en hiver, au printemps et en été et analysés pour certains paramètres hématologiques. Les globules blancs (WBC) étaient plus élevés au printemps et en été, ce qui peut être lié à la promiscuité sexuelle. Les moineaux étaient plus stressés en été. Les globules rouges (GR) et l“hématocrite (Het) étaient plus élevés en été. Le volume corpusculaire moyen (MCV) est plus faible en été. Cela peut avoir une adaptation pour faire face à un stress élevé en été, car les globules rouges de petite taille augmentent les échanges gazeux. Les plaquettes n”étaient pas affectées par la saison ou le sexe. Le MCV et l“hémoglobine corpusculaire moyenne (MCH) sont positivement corrélés l”un à l“autre. Les globules rouges, l”hémoglobine (Hb) et le MCV étaient plus élevés chez les mâles au printemps, peut-être en raison d“une adaptation aux activités énergétiques au printemps comme les cris d”accouplement et la recherche de sites de nidification. Les globules blancs sont restés les mêmes chez les deux sexes en été et en hiver, et effectués au printemps peuvent être liés au système d“accouplement. L”état comportemental est lié aux états physiologiques qui montrent des traits de compromis et d'histoire de vie. Cette étude est un petit effort pour connaître cette espèce incroyable. Nous pouvons continuer à travailler dans différentes parties du monde pour en explorer différents aspects.

## Introduction

*Passer domesticus* is a globally adaptive bird. It can be found breeding in hot places like deserts in southern California or living in cold areas of the Arctic Circle. (Hanson et al. 2020). It shows that it has adapted to live in extreme environments. The house sparrow can tolerate intense fluctuations. Annual seasonal variations are important environmental factors in an area. House Sparrow also adapted to live in the fluctuations and variations during seasonal change. Pakistan has four seasons in a year, sparrows need to adapt to each season accordingly. The purpose of this study is to investigate seasonal variation in the hematological parameters of house sparrows in Pakistan.

Seasonal changes are a very important factor in studying behaviors and adaptations. It not only alters the environment around but inside of a living being too. There are two ways an animal manages. Either changing its internal environment to cope with the external conditions or changing its behavior. Either or adapted increases its fitness. Let's take a look into some of the seasonal variations and adaptations in some birds. During winter and autumn, the food storage behavior increases because of the shortage of food. In some birds like chickadees, tits, jays, nutcrackers, and nuthatches, size of the hippocampus increases as their adaptation (Sherry and Hoshooley 2010). Just like an increase in hippocampus size, some temperate birds increase their immunity during breeding times and they do so by increasing the size of the spleen and immune cells (Moller et al. 2003) because during breeding chances of getting disease increase. This is how the seasons are affecting physiology and behaviors. Those physiological and behavioral changes may have adapted that are supported by these changes. The behavioral state is linked with the physiological state that shows tradeoffs and life history traits. During winter, birds face thermoregulatory problems, especially during cold nights. For surviving cold winter, hemoglobin (Hb) concentration (Swanson 1990), basal metabolic rate, and body mass (Liknes and Swanson 1996) change. The dark-eyed Junko meets its thermoregulatory demand by increasing hematocrit (Hct) values and oxygen capacity in winter Swanson DL (Swanson 1990). Seasonal changes affect both immunity (Kelly et al. 2018) and Hb concentration (Haas 2020) in birds. We are studying the effect of seasonal variations on hematological parameters. The hematological parameters are physiological indicators and provide information regarding the physiology and ecology of the organism. Summer is hot and dry. Rearing of offspring may also add to the stress on House sparrow. During spring, the food availability is high along with the start of the breeding season. Breeding and reproduction are high energy-demanding activities. In adult house sparrow, Hb concentration reportedly increases in summer (Puerta et al. 1995). The size of the erythrocytes elevates during winter in the alpine accentor (Passeriformes) and vice versa in summer (Janiga et al. 2017).

The parameters differ during different stages of the life cycle. It may also indicate stress (Llacuna et al. 1996) and water shortage (Puerta et al. 1995). Hematocrit and Hb are plastic traits (Yap et al. 2019) and directly relate to metabolic performance (Butler 2016). Environmental conditions, habitat (Herrera-Duenas et al. 2014) and dehydration are the main factors affecting Hb concentration (Kasprzak et al. 2006). During the migration of birds, values of Hct changes to increase fitness for successful migration (Krause et al. 2016; Butler 2016). This is how hematological parameters differ as an adaptation for surviving in changing circumstances. Hematological factors and immune functions are not only altered by changes in environmental conditions but adults and immature also differ in these factors (Kasprzak et al. 2006). These factors also alter according to the nutritional stage of the bird (Kelly et al. 2018). So for assessing hematological parameters’ changes in different seasons, we need to keep in mind these factors too. For the successful rearing of offspring, physiological and behavioral changes occur between breeding and non-breeding seasons, for instance, changes in Hb concentration (Velmala et al. 2015) and blood oxygen capacity. These values increase by 18% during spring in *Malurus cyaneus* (Australia).

These adaptations are the product of millions of years of evolution. These traits increase the survival and reproductive fitness of organisms and give information about the changing behavioral patterns concerning ecology. The study is focusing on the scientific information of how avian species interact with ecological factors like seasonal variations. Therefore, this study investigated phenotypic plasticity in house sparrow in response to seasonal changes. Hematological parameters change with seasons as tradeoffs between these parameters and energy requirements in house sparrows. We are testing our hypothesis that seasonal flexibility in hematological parameters is an adaptive trait and helps house sparrow adapts to changing circumstances. The study will give indices for hematological parameters of the house sparrow seasonally and knowledge about adaptive traits and phenotypic plasticity in house sparrows.

## Methodology

Seasonal variations in hematological parameters are used as an experimental approach to study the differences in the physiology of sparrows in each season and its effect on the behaviors and overall fitness of house sparrows. We captured house sparrows (adults) in winter, spring, and summer from the same local population of the sub-tropical area, Potohar Punjab, Pakistan. The male is having gray-colored head and black bib. The female is brown colored dull in appearance as compared to the male, but its plumage is darker than juvenile. The young sparrow's beak color is light brown. The adult has a dark-colored beak (Anderson 2006). During winter, we captured sparrows in February 2022, during spring (early breeding) in April 2022, and in summer (late breeding) in July 2022. In the Potohar region, winter is roughly from December to February, spring in March and April, and Summer from round about May to August. Every time we released sparrows soon after blood sampling. Ten percent of total blood volume can be taken from the bird (Kramar 2015) or 1% of the body weight (Joslin 2009). Two hundred and fifty microliter (μL) to 300 μL (0.25 milliliter (ml) to 0.30 ml) blood was taken from the jugular vein. (Most samples were taken from the jugular vein. However, some samples were taken from the branchial or wing vein (Kramer and Harris 2010) by using 30-gauge BD ultra-fine. After collection, the blood was transferred to an EDTA solution tube. That contained 45 μL solution of EDTA and stored at 4°C before hematological analysis. Fifteen, Eleven, and Twelve samples were taken in winter, spring, and summer, respectively. Out of them, eight were male and seven were female in winter. During spring, five males and six Females were captured. Six males and females were taken in the summer. The tested blood parameters include Red blood cell (RBC) counts, White blood cell (WBC) counts, platelet counts, Hb concertation, Hct, Mean corpuscular volume (MCV), Mean corpuscular hemoglobin (MCH), Mean corpuscular hemoglobin concentration (MCHC), and differential leukocyte counts (Heterophill, lymphocytes, monocytes, eosinophils, and basophils). The blood tests were performed on a hematology machine Urate Hematology Analyzer veterinary version model 3000 plus Germany. A factorial design analysis of variance followed by Tukey's test and correlation are used for statistical analysis. The value of *P* < 0.05 is significant. MSTAT-C 1988, Michigan State University, MI, USA was used for statistical analysis.

## Results

### Seasonal changes in hematological parameters

WBCs: We found that seasonal changes are affecting studied immune parameters WBCs (*P *= 0.001), Monocytes (*P *= 0.00), Eosinophils (*P *= 0.00), Heterophils (*P *= 0.00), and Lymphocytes (*P *= 0.00). The H/L ratio is also affected by seasons (*P *= 0.00) and is found highest in summer. Monocytes and Eosinophils were found least in winter. Heterophils were highest in summer, and Lymphocyte was highest in winter. Lymphocytes and heterophils were negatively correlated with each other in all seasons (see Table 2). Heterophils are highest in summer and least in winter. Lymphocytes are least in summer and highest in winter. This gave a H/L ratio to be greatest for summer. It may suggest more stress in the summer season. Basophils are not affected by season (*P *= 0.1063).

RBCs: Seasons impart effect on RBCs (*P *= 0.0004). It was found highest in summer.

Platelets: Platelets (*P *= 0.295) have not affected by season. During winter and summer, WBCS and Platelets were positively correlated but in spring negative correlation was found (see Table 2).

Hemoglobin: Seasons haven't affected Hemoglobin concentration (Hb) (*P *= 0.054). Hb and RBCs have a positive correlation in all seasons (see Table 2).

Hematocrit: Hematocrit was affected by seasons (*P *= 0.00). It was found highest in summer and least in winter. RBCs and Hct have a positive correlation in all seasons (see Table 2).

MCH, MCV, and MCHC: Values of MCH (*P *= 0.00), MCV (*P *= 0.00), and MCHC (*P *= 0.00) were affected by seasons. MCV and MCH were highest in winter and least in summer. MCHC was highest in spring and lowest in winter. RBCs and MCV were negatively correlated in summer and winter and positively correlated in the spring season. MCH and MCV have a positive correlation in all seasons. MCHC and MCV were positively correlated in spring and summer and a negative correlation was found in the winter. MCHC and MCH have a positive correlation in summer and spring and a negative correlation in winter season. RBCs and MCH are negatively correlated in winter and positively correlated in spring and summer (see Table 2)

### Gender differences in hematological parameters

All hematological parameters differed between both genders except Platelets (*P *= 0.139) and Basophils (0.073).

During winter, Hb, Hct, and MCH were found higher in females (see Table 1). Both genders have a positive correlation between MCH and Hb (see Table 2).

**Table 1:** Seasonal and gender-based variations in hematological parameters of house sparrow

S. No	Hematological parameters		Winter	Spring	Summer	Total
1	WBC (counts/uL)	Male	43,412.5 ± 8070^bc^	56,050 ± 9800^ab^	55,333 ± 1190^a^	50,780 ± 10,884*
		Female	39,300 ± 1155^bc^	60,040 ± 6540^c^	48,841.6 ± 1421^ab^	48,241 ± 13,806
		Total	41,493.3 ± 9712^w^	57,863.6 ± 8335^u^	52,087.5 ± 12948^u^	
2	RBC (counts M/uL)	Male	2.9 ± 0.24^bc^	3.32 ± 0.29^ab^	3.4 ± 0.402^a^	3.225 ± 0.36*
		Female	2.94 ± 0.34^bc^	2.61 ± 0.42^c^	3.2 ± 0.397^ab^	2.9 ± 0.43
		Total	2.95 ± 0.28^w^	3.0 ± 0.50^w^	3.37 ± 0.40^u^	
3	Platelets (count/uL)	Male	41,500 ± 7874	46,500 ± 6220	60,833.3 ± 12528	48,800 ± 12,007
		Female	48,428 ± 8618	32,000 ± 5338	46,500 ± 1187	43,222 ± 11,243
		Total	44,733.3 ± 8697	39,909 ± 9385	53,666 ± 13838	
4	Hemoglobin (g/dL)	Male	13.9 ± 1.1^bc^	15.4 ± 0.63^ab^	15.9 ± 1.32^a^	14.97 ± 1.35*
		Female	15.9 ± 1.39^a^	12.3 ± 1.32^c^	12.7 ± 1.81^c^	13.85 ± 2.23
		Total	14.8 ± 1.59	14 ± 1.88	14.32 ± 2.26	
5	Hematocrit (%)	Male	34.18 ± 2.92^c^	42 ± 2.51^ab^	46.6 ± 4.8^a^	40.28 ± 6.35*
		Female	39.7 ± 4.62^b^	39 ± 5.77^bc^	41.1 ± 3.7^b^	39.9 ± 4.49
		Total	36.76 ± 4.64^w^	40.6 ± 4.35^uw^	43.9 ± 5^u^	
6	MCV (fL)	Male	123.8 ± 625^abc^	128.4 ± 5.66^a^	115.1 ± 8.2^d^	122.5 ± 8.42*
		Female	125.7 ± 3.91^ab^	117.9 ± 6.44^bcd^	109.6 ± 7.8^d^	118.2 ± 9.09
		Total	124.7 ± 5.37^u^	123.6 ± 7.9^u^	112.3 ± 8.15^w^	
7	MCH (pg)	Male	49.2 ± 2.60^b^	47.6 ± 2.188^bc^	44.9 ± 2.50^c^	47.4 ± 2.95
		Female	53.6 ± 2.13^a^	46.5 ± 2.42^bc^	41.2 ± 3.34^d^	47.55 ± 6.02*
		Total	51.3 ± 3.25^u^	47.1 ± 2.25^w^	43 ± 3.43^x^	
8	MCHC (g/dL)	Male	46.2 ± 3.88	51.8 ± 1.98	49.4 ± 3.90	48.8 ± 4.02*
		Female	45.3 ± 0.93	51.2 ± 0.92	46.2 ± 2.37	47.28 ± 2.96
		Total	45.84 ± 2.85^w^	51.5 ± 1.52^u^	47.8 ± 3.48^w^	
9	Heterophil (%)	Male	7.8 ± 2.1^c^	8.6 ± 1.50^bc^	12.3 ± 4.03^a^	9.54 ± 3.21*
		Female	7.1 ± 1.86^c^	9.4 ± 1.34^abc^	11.1 ± 2.04^ab^	9.1 ± 2.44
		Total	7.53 ± 1.95^w^	9 ± 1.4^w^	11.75 ± 3.10^u^	
10	Lymphocyte (%)	Male	86.6 ± 2.66^a^	83 ± 2.6^b^	78.33 ± 4.03^d^	83.05 ± 4.59
		Female	88.1 ± 2.26^a^	81.2 ± 1.09^bc^	79.83 ± 0.983^cd^	83.4 ± 4.1*
		Total	87.3 ± 2.52^u^	^z^82.1 ± 2.18^w^	79 ± 2.9^x^	
11	Monocyte (%)	Male	2.75 ± 0.70	4 ± 1.26	3.6 ± 1.032	3.4 ± 1.09
		Female	2.2 ± 0.48	4.2 ± 0.83	4.33 ± 0.816	3.5 ± 1.20*
		Total	2.53 ± 0.63^w^	4.09 ± 1.04^u^	4 ± 0.95^u^	
12	Eosinophil (%)	Male	2 ± 0.53	3.83 ± 0.98	4.67 ± 1.211	3.35 ± 1.46
		Female	2.1 ± 0.69	4.6 ± 0.54	4.33 ± 1.033	3.55 ± 1.38*
		Total	2.06 ± 0.59^w^	4.18 ± 0.87^u^	4.5 ± 1.08^u^	
13	Basophil (%)	Male	0.62 ± 0.51^ab^	0.50 ± 0.54^ab^	1 ± 0.63^a^	0.7 ± 0.57
		Female	0.28 ± 0.48^b^	0.6 ± 0.54^ab^	0.50 ± 0.548^ab^	0.44 ± 0.51
		Total	0.46 ± 0.51	0.54 ± 0.52	0.75 ± 0.62	
14	H/L ratio	Male	0.09 ± 0.027	0.103 ± 0.01	0.16 ± 0.06	0.109 ± 0.05
		Female	0.081 ± 0.023	0.115 ± 0.01	0.14 ± 0.026	0.11 ± 0.03*
		Total	0.079 ± 0.031^u^	0.108 ± 0.018^uw^	0.24 ± 0.03^w^	

*Note:* (Tukey test, values with similar alphabets are statistically similar, for seasons u, w, x and for gender and season interaction a, b, c *P* < 0.05 significant).

(Gender differences during each season is indicated by * *P *< 0.05.)

**Table 2:** Correlation for some hematological parameters in all seasons (*P*-values were corrected for multiple testing of correlation using the Benjamini–Hochberg procedure at α = 0.05) (Adjusted *P* value = Adj *P*)

		Winter			Spring			Summer		
S. No	Correlations	*r* ^2^	*P*	Adj *P*	*r* ^2^	*P*	Adj *P*	*r* ^2^ value	*P*	Adj *P*
1	WBCs—Platelets	0.193	0.491	0.936	−0.21	0.519	0.519	0.54	0.067	0.168
2	RBCs—Hb	0.40	0.137	0.474	0.75	0.007	0.038	0.33	0.280	0.514
3	RBCs—Hct	0.38	0.151	0.474	0.29	0.384	0.470	0.26	0.406	0.558
4	RBCs—MCV	−0.19	0.494	0.766	0.54	0.082	0.182	−0.10	0.751	0.751
5	RBCs—MCH	−0.04	0.881	0.955	0.22	0.499	0.519	0.15	0.626	0.751
6	MCV—Hb	0.16	0.557	0.766	0.84	0.001	0.012	0.64	0.024	0.091
7	MCV—MCH	0.37	0.172	0.474	0.67	0.023	0.085	0.29	0.352	0.553
8	MCH—Hb	0.25	0.353	0.647	0.44	0.172	0.271	0.52	0.076	0.168
9	MCHC—MCV	−0.01	0.955	0.955	0.51	0.101	0.185	0.70	0.011	0.061
10	MCHC—MCH	−0.02	0.922	0.955	0.33	0.312	0.429	0.11	0.732	0.751
11	Hetero—Lympho	−0.94	0.000	0.000	−0.61	0.043	0.120	−0.82	0.001	0.011

During spring, RBCs, Hb, and MCV were found to be higher in males (see Table 1).

During summer, Hb, Hct, and MCH were different in both genders. It was higher in males (see Table 1).

Effect of both Season and Gender: RBCs (*P *= 0.0043), Platelets (*P *= 0.00), Hb (*P *= 0.00), Hct (*P *= 0.0003), MCV (*P *= 0.0172), MCH (0.0001), Heterophil ((*P *= 0.00), Lymphocyte (*P *= 0.023), and Basophils (*P *= 0.00) are affected by both season and gender.

## Discussion

The seasonal variations in Hematological parameters reflect the physiological condition. They also differ during different stages of the life cycle and different seasons.

In our study, hematological parameters showed seasonal variation. However, in the current study, hemoglobin concentration (Hb) platelets and basophils remained the same in all seasons. However, its values reputedly changed in free-living pigeons (*Columba livia f. urbana*). Hemoglobin and Hct values differ during different phases of the life cycle. Laying, pause laying, and molting periods (Kasprzak et al. 2006). However, in our case, it remained same. It indicates that hemoglobin is affected by life stages but not seasonal changes.

A study in Spain reported mean WBC counts of 21,800 ± 2400 cells/mm^3^ for male house sparrows during June (Puerta et al. 1995). This value of WBCs is lower than our house sparrows’ mean WBCs during summer (July month). Literature suggests birds may trade immunity for molting during winter (Nava et al. 2001). It supports our findings of lower WBCs in winter. During the study, we found higher WBCs in spring. Birds breeding activities during spring and summer may make them more vulnerable to disease. So, it seems that the increase in WBCs during spring was the response to the exposure to infection. Pieces of Evidence from research on primates have suggested that immune parameters had direct relations with the mating systems in promiscuous animals, and WBCs were higher (Nunn 2002; Nunn et al. 2000). Though, the house sparrow is a socially monogamous bird species. Its social monogamy does not match genetic monogamy. Both male and female house sparrows may be mating with extra-pair individuals. Mating takes place throughout the breeding season and many times a day. It may be the reason for greater WBC counts during the spring and summer in the current study.

In our study, higher H/L ratio in summer suggests more stress in sparrows during the summer. A study on reed buntings in Poland showed a higher H/L ratio for spring (breeding season) than for summer (post-breeding). They concluded that the H/L ratio is independent of sex (Jakubas et al. 2011). Another study in Western Poland on White Stork *Ciconiaciconia* chicks found a positive correlation of H/L ratio with Heterophil and a negative correlation with lymphocytes. WBCs have a positive correlation with the H/L ratio (Kaminski et al. 2014). These correlations are as per our findings. Platelets were found highest in summer and least in spring in the present study. Platelets represent infection rate (chances of disease and injury may cause an increase in platelets) to infections. In case of any viral or bacterial infection, activate the immune system. Platelets are reportedly found to have functions in innate and adaptive immunity (Assinger 2014). Mostly its immune role has been neglected. It protects the host from infection, like lymphocytes. It activates the immune system.

According to our findings, Hct was found higher in summer and least in winter, possibly due to shortage of water during summer. Water shortage decreases blood plasma volume. RBCs and Hct have positive correlation in all seasons. Onyeyili et al. (1991) reported positive correlation between RBCs and Hct in Guinea fowl. Previously some authors reported Hct values in male house sparrow (39.4%), adult pigeon guillemots (58%), and wild blue rock pigeons (*Columbalivia*) (49.3%) (Puerta et al. 1995; Seiser et al. 2000; Khan et al. 2011). Literature suggests that Hct is affected by many factors like energy requirements, nutrition, age, higher altitudes, etc. It increases with elevation but not always (Fair et al. 2007). In case of house sparrow, elevation cannot be a reason because it remains on the same elevation throughout its life time and not a migratory bird.

Based on our findings, MCH and MCV values were higher in winter and least in summer. MCHC value was significantly highest in spring and least in winter. Kaminski et al. (2014) studied White Stork (*Ciconia ciconia*) chicks during breeding season (March and April). They find a positive correlation between Hb and RBCs. MCH has a positive correlation with MCHC and Hb. MCH and MCV are positively related (similar to our findings). MCV had a negative relation with Hb, Hct, and RBCs. MCV and MCHC are negatively related (contradictory to our results). Hawkey et al. (1991) found a positive correlation between MCV and MCH. And a strong negative correlation between RBCs and MCV in mammals, birds, and reptiles from 110 families. We found a similar correlation between RBCs and MCV except in spring, where we found a positive correlation. The mean MCH, MCV, and MCHC were previously reported for wild blue rock pigeons (*Columba livia*). Their calculated MCH mean value (45.9 ± 13.7) was equal to our calculated values. The MCV and MCH mean values were lower than our study values (Khan et al. 2011).

Weathers and Sullivan calculated field metabolic rate (FMR) in yellow-eyed juncos and dark-eyed juncos during breeding and non-breeding seasons. They found, no significant difference in FMR in both seasons. They found winter to be more energy demanding (Weathers and Sullivan 1993). In present study, RBC counts and Hct have positive correlation. It strongly suggests seasonal difference in plasma volume. MCV and MCH were significantly higher in winter. They had positive correlation with each other. It may support the above argument that winter require more energy for temperature maintenance. Janiga et al. (2017) worked on alpine accentors and reported the erythrocyte size to be largest in winter (greater MCV) and smallest in summer (smaller MCV). It is similar to my findings. They reported that smaller RBCs are found during start and end of breeding season and comparatively larger sized RBCs in middle of breeding season. This suggests smaller erythrocyte size during breeding season. Breeding is an energy demanding activity. Small size RBCs have large surface area to volume ratio that allow greater gas exchange during breeding to fulfill higher energy requirements. These studies’ results support present study seasonal variations of MCV and MCH.

RBCs, Hb, and MCV were found significantly higher in male during spring. Erythrocyte number, size (MCV), and Hb concentration are linked with energetic life style (Soulsbury et al. 2021). It can be related to the behaviors of male house sparrow. The life history traits shaped physiology and behavior in accordance with each other. In spring, during start of breeding season, male finds nesting place producing mating calls. All these are energy demanding activities and require high oxygen supply. Physical condition of body plays an important role. The sex hormones affect erythropoiesis process. The androgens accelerate it, and estrogens decreases it (Puerta et al. 1995). This may be the reason of higher RBCs number in male house sparrows in our study. Kilgas et al. (2006) studied on great tit haematology. They find heterophil and H/L ratio differ in both sexes. They find female health poorer and having higher H/L ratio than male during breeding season. When great tits were rearing their first brood, they had higher heterophil and lymphocyte values. During second brood rearing, these values decreased. They further studied that males with high lymphocytes had greater survival. In our study of house sparrows, a positive correlation was found between RBCs and Hb concentration during the spring season. A positive correlation was found between Hb and MCV in both genders. Males had a positive correlation between RBCs and MCV, and females had a negative correlation. Captive Adult Black-faced Ibis (*Theristicus melanopis melanopis*) hematological parameters were studied in Chile. They found no significant difference in RBC and MCV in both genders (Silva et al. 2020).

While comparing males and females in spring, significant difference was found in WBCs count. Moller et al. (1996) studied that during the breeding season, males with high secondary sexual characters have small bursa of Fabricius (less investment to immunity). According to Moller, WBC counts should be less in males. Testosterone decreases immune functions as a tradeoff between immunity and sexual character. In polygamous species, the male has greater competition and high levels of testosterone that causes fewer immune cells as compared to monogamous male (Klein 2000), similar to our study. According to Valdebenito et al. (2021), immune differences in sexes are more in the breeding season. This case is similar to our study.

Hemoglobin was found higher in males during summer. It was found to be greater in males. Testosterone causes an increase in Hb levels (Puerta et al. 1995). Our results during summer suggest that seasonal variations may affect both genders similarly, but their behavioral differences and physiological state cause a tradeoff between some parameters. Hematology and its relation to fitness-related traits have been the focus of this study. This study gave some findings regarding the behavioral ecology of the house sparrow and how it has adapted to seasonal changes in an ecosystem. It proved our hypothesis that seasonal flexibility in hematological parameters is an adaptive trait and helps house sparrows adapt to changing circumstances. More study is required for a better perceptivity of the house sparrow's physiology and its relation to its behaviors, we lack research in this field, and the scope of the study can be expanded by adding more hematological and biochemical parameters. Taking data from different ecological zones and comparing them will make it more interesting.

## Limitations

This study was focused on seasonal variations of hematological parameters. However, the study does not include data for autumn. Moreover, the small sample size is another limitation.

## Supplementary Material

obad027_Supplemental_File
