# Supplementary material for: Seasonal Changes in Hematological Parameters in House Sparrows of Subtropical Pakistan
Source: Integr Org Biol. 2023 Jul 26;5(1):obad027. doi: 10.1093/iob/obad027 (PMC10399915; doi:10.1093/iob/obad027)
Supplement: obad027_Supplemental_File [file obad027_supplemental_file.docx]

**Supplementary:**

**Table 1** Results of RBCD, Factorial Design. (A= Gender, B=Seasons, AB= interaction of gender and season)

| **Hematological Parameters** | WBCs | RBCs | Platelets | Hb | Hct | MCV | MCH | MCHC | Het | Lymp | Mono | Eos | Baso | H/L ratio |
| --- | --- | --- | --- | --- | --- | --- | --- | --- | --- | --- | --- | --- | --- | --- |
| **AB** | 0.199 | 0.0043 | 0.00 | 0.00 | 0.0003 | 0.0172 | 0.0001 | 0.279 | 0.00 | 0.023 | 0.205 | 0.096 | 0.00 | 0.214 |
| **A** | 0.00 | 0.0002 | 0.139 | 0.0002 | 0.00 | 0.0093 | 0.00 | 0.032 | 0.00 | 0.00 | 0.00 | 0.00 | 0.073 | 0.00 |
| **B** | 0.00 | 0.0004 | 0.295 | 0.054 | 0.00 | 0.00 | 0.00 | 0.00 | 0.00 | 0.00 | 0.00 | 0.00 | 0.1063 | 0.00 |
| **F-value** | 1.683 | 6.393 | 0.2623 | 24.93 | 10.3 | 4.57 | 12.5 | 1.32 | 0.933 | 4.19 | 1.655 | 2.50 | 0.693 | 1.61 |
| **df** | 2 | 2 | 2 | 2 | 2 | 2 | 2 | 2 | 2 | 2 | 2 | 2 | 2 | 2 |
| **Denominator df** | 35 | 35 | 35 | 35 | 35 | 35 | 35 | 35 | 35 | 35 | 35 | 35 | 35 | 35 |
